# Impact of ACO enrollment on prescription drug costs for Medicare FFS beneficiaries newly diagnosed with ADRD

**DOI:** 10.1093/geroni/igaf122.3181

**Published:** 2025-12-31

**Authors:** Seyeon Jang, Jie Chen

**Affiliations:** University of Maryland School of Public Health, College Park, Maryland, United States; University of Maryland College Park, College Park, Maryland, United States

## Abstract

Medications are a cost-effective treatment for managing chronic conditions and reducing downstream medical costs. Accountable Care Organizations (ACOs) can enhance medication adherence through improved coordination and integration of care, promote the substitution of brand-name drugs with generics, engage pharmacists in direct patient care, and educate patients on therapeutic alternatives. This study examines the effects of ACO enrollment on Part D prescription drug costs among Medicare beneficiaries newly diagnosed with Alzheimer’s Disease and Related Dementias (ADRD). We used a 2016–2022 longitudinal cohort dataset integrating the Medicare Beneficiary Summary File, MBSF Cost and Use, and Medicare Shared Savings Program ACO beneficiary-level files. The study included 111,235 beneficiaries newly diagnosed with ADRD in 2017 who remained in Medicare fee-for-service throughout the study period. Generalized estimating equation models were applied, controlling for individual- and neighborhood-level characteristics. Our findings show that prescription drug costs for newly diagnosed ADRD patients increased over time. While ACO enrollees without multiple chronic conditions (MCC) had consistently higher predicted drug costs than non-ACO counterparts, those with MCC encountered progressively growing cost savings, from $55 in 2016 to $93 in 2022, and those with multisystem morbidity (five or more chronic conditions), from $70 to $119. These findings suggest that ACO participation may help lower medication costs for patients with complex health needs while improving care coordination. Further incentives are needed to encourage ACOs to take a more proactive role in managing prescription drug use, given its significant contribution to reducing overall healthcare costs.

